# The structure of metallo-DNA with consecutive thymine–Hg^II^–thymine base pairs explains positive entropy for the metallo base pair formation

**DOI:** 10.1093/nar/gkt1344

**Published:** 2013-12-26

**Authors:** Hiroshi Yamaguchi, Jakub Šebera, Jiro Kondo, Shuji Oda, Tomoyuki Komuro, Takuya Kawamura, Takenori Dairaku, Yoshinori Kondo, Itaru Okamoto, Akira Ono, Jaroslav V. Burda, Chojiro Kojima, Vladimír Sychrovský, Yoshiyuki Tanaka

**Affiliations:** ^1^Laboratory of Molecular Transformation, Graduate School of Pharmaceutical Sciences, Tohoku University, 6-3 Aza-Aoba, Aramaki, Aoba-ku, Sendai, Miyagi 980-8578, Japan, ^2^Institute of Organic Chemistry and Biochemistry, Academy of Sciences of the Czech Republic, Flemingovo nám. 2, 166 10, Praha 6, Czech Republic, ^3^Department of Materials and Life Sciences, Faculty of Science and Technology, Sophia University, 7-1 Kioicho, Chiyoda-ku, Tokyo 102-8554, Japan, ^4^Department of Material and Life Chemistry, Faculty of Engineering, Kanagawa University, 3-27-1 Rokkakubashi, Kanagawa-ku, Yokohama, Kanagawa 221-8686 Japan, ^5^Department of Chemical Physics and Optics, Faculty of Mathematics and Physics, Charles University in Prague, Ke Karlovu 3, 121 16 Prague 2, Czech Republic and ^6^Institute for Protein Research, Osaka University, 3-2 Yamadaoka, Suita, Osaka 565-0871, Japan

## Abstract

We have determined the three-dimensional (3D) structure of DNA duplex that includes tandem Hg^II^-mediated T–T base pairs (thymine–Hg^II^–thymine, T–Hg^II^–T) with NMR spectroscopy in solution. This is the first 3D structure of metallo-DNA (covalently metallated DNA) composed exclusively of ‘NATURAL’ bases. The T–Hg^II^–T base pairs whose chemical structure was determined with the ^15^N NMR spectroscopy were well accommodated in a B-form double helix, mimicking normal Watson–Crick base pairs. The Hg atoms aligned along DNA helical axis were shielded from the bulk water. The complete dehydration of Hg atoms inside DNA explained the positive reaction entropy (Δ*S*) for the T–Hg^II^–T base pair formation. The positive Δ*S* value arises owing to the Hg^II^ dehydration, which was approved with the 3D structure. The 3D structure explained extraordinary affinity of thymine towards Hg^II^ and revealed arrangement of T–Hg^II^–T base pairs in metallo-DNA.

## INTRODUCTION

The metal-mediated base pairs (the metallo base pairs) are currently being explored toward genetic code expansion (1–4), development of metallo-DNAs (5–12), molecular magnets (13,14), electric nano-wires (15–19) and metal ion-sensors (20,21). Among these, the Hg^II^-sensor employing thymine–Hg^II^–thymine (T–Hg^II^–T) base pair was the first successful application (20).

The success of this Hg^II^-sensor was owing to both the extraordinary Hg^II^-thymine specificity and the thermal stability of T–Hg^II^–T base pair (22–25). The thermal stability of the T–Hg^II^–T base pair was similar as those of normal Watson–Crick (W–C) base pairs (24). Moreover, the positive Δ*S* recorded for T–Hg^II^–T base pair formation with isothermal titration calorimetry (ITC) (24) indicated its peculiarity, since biomolecular complexations are usually linked with negative Δ*S* values (26,27). However, the lack of structural data for T–Hg^II^–T base pairs in a DNA duplex prohibited rational explanation of this positive Δ*S*. In addition, as apparent from structure-based drug designs, the explanation of Δ*S* on structural basis is difficult and rarely possible. Therefore, the elucidation of entropic contributors from three-dimensional (3D) structures is challenging issue in structural biology/chemistry.

The metallo-DNA with T–Hg^II^–T base pairs is regarded promising conductive nano-material. Several groups examined its ability to mediate hole/electron transport, and weak-hole transport similar to that in normal DNA was actually observed (15–18). However, as mentioned above, the lack of structural information for T–Hg^II^–T pairs prevented rationalization of these experiments and tuning of conductivity in metallo-DNAs.

The binding mode of Hg atom in T–Hg^II^–T base pair was surely determined using the ^15^N NMR and Raman spectroscopy (23,28,29). The theoretical calculations based on its structure suggested that the LUMOs appearing around Hg^II^ of the T–Hg^II^–T base pair is distributed along the DNA-helical axis (29). Therefore, it is important to reveal mutual positioning of T–Hg^II^–T base pairs in the metallo-DNA and to confirm if the overlap of their LUMOs is possible or not.

The metallophilic attraction between Hg atoms in consecutive T–Hg^II^–T base pairs stabilizes structure of metallo-DNA although the Hg atoms in these metallo base pairs bear sizable positive charge (29–31). Only few observations of the metallophilic phenomenon were reported so far for some organometallic complexes. The 3D structure of metallo-DNA would provide reliable basis for physicochemical investigation of Hg–Hg metallophilic attraction.

The 3D structures of metallo-DNAs, which are currently available include solely those composed of ‘ARTIFICIAL bases’ (metal-chelators) (1,2,11). The structural information on metallo-DNAs composed of ‘NATURAL bases’ is therefore very sparse. Only the T–Hg^II^–T base pair was thoroughly studied with molecular spectroscopy (23,28,29,32–36), 3D modeling (34) and the crystal structure of 1-methylthymine–Hg^II^ complex (37). Although the binding mode of Hg^II^ was determined in these studies, 3D structure of the metallo-DNA duplex remained unresolved.

The lack of structural information for the metallo-DNA duplex in solution made us initiate this study aiming particularly explanation of the thermodynamic parameters for T–Hg^II^–T base pair formation.

## MATERIALS AND METHODS

### Thermal denaturation experiment

**Figure 1.**
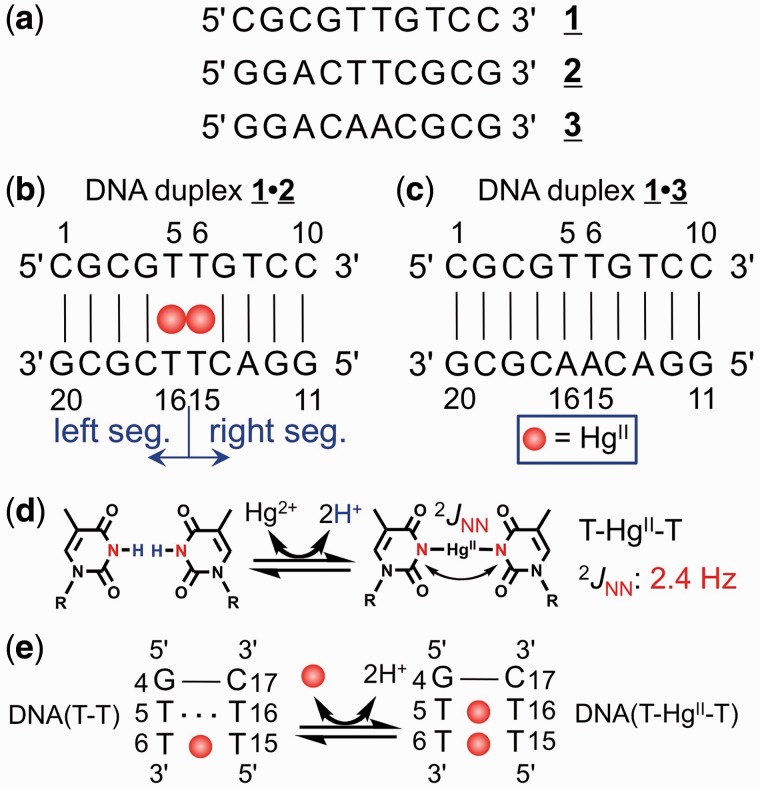
The DNA sequences and the T–Hg^II^–T base pair. (**a**) The DNA oligomers **1**, **2** and **3**. (**B**) The DNA duplex **1**•**2** with residue numbers. The definition of left and right segments is depicted. (**c**) The control DNA duplex **1**•**3** with residue numbers. (**d**) The reaction scheme for T–Hg^II^–T base pair formation (the proton–Hg^II^ exchange reaction) and 2-bond ^15^N–^15^N *J*-coupling (^2^*J*_NN_) (23). (**e**) The schematic representation of the model used in ONIOM QM/QM calculation of thermodynamic parameters. The DNA(T–T) and DNA(T–Hg^II^–T) stand for Hg^II^-free and Hg^II^-bound three base-paired (3 bp) duplex.

**Figure 2.**
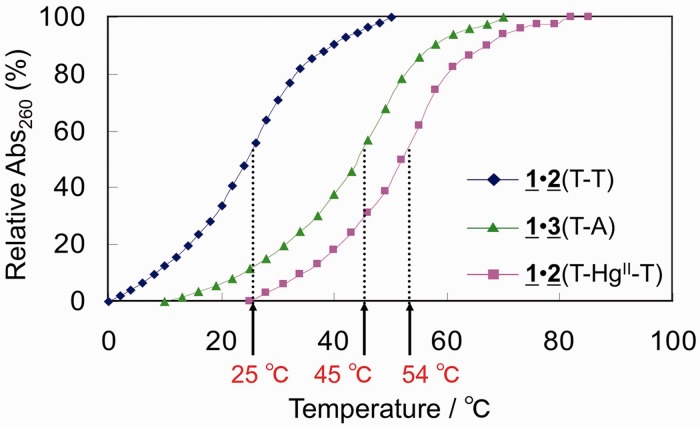
The temperature profiles of UV absorbance at 260 nm. The vertical axis is a relative absorbance normalized between absorbances at the lowest and the highest temperatures. Blue diamonds: the DNA duplex **1**•**2** in the absence of Hg^II^. Pink squares: the DNA duplex **1**•**2** in the presence of Hg^II^. Green triangles: the DNA duplex **1**•**3**. The *T*_m_ values for these profiles are given by the red characters with arrows.

DNA sequences used for this experiment are listed in Figure 1 and Supplementary Figure S1. UV spectra of the solutions of DNA decamers were recorded every 3°C for ‘**1**•**2**(T–Hg^II^–T)’ and ‘**1**•**3**(T – A)’, and every 2°C for ‘**1**•**2**(T–T)’ (Figure 2). In the temperature profiles, UV absorbances at 260 nm were plotted against temperature (Figure 2). The *T*_m_ value which is dependent on the nearest neighbour W–C base pairs against T–Hg^II^–T base pairs was studied using a dodecamer hetero duplex: d(CCGC**X**TT**V**TCCG) • d(CGGA**W**TT**Y**GCGG), where **X**–**Y** and **V**–**W** are W–C base pairs (Supplementary Figure S1). The effect of concentration of Hg^II^-bound duplex **1**•**2** on *T*_m_ value was also examined (Supplementary Figure S2). The *T*_m_ values were determined using a method described in the literature (38). In all the thermal denaturation experiments, we confirmed that temperature profiles for increasing and decreasing the temperatures were identical within the experimental error range. For further details, see Supplementary Material.

### NMR measurements and 3D structure determination

NMR spectra for the ^1^H resonance assignments and structure calculations were measured as described previously (23,39). By using the derived NMR spectra, we assigned all the non-exchangeable protons (39) and most of the exchangeable protons. The complete assignments are reported in Supplementary Table S1, and deposited in the Biological Magnetic Resonance Data Bank with accession number 11 528. Resulting experimental constraints and other constraints for structure calculations are listed in Supplementary Table S2. The structural constraints for the T–Hg^II^–T base pairs were generated based on the crystal structure of the 1-methylthymine–Hg^II^ (2:1) complex (37), i.e. N3–Hg^II^ bond length: 2.04Å and N3–Hg^II^–N3-bond angle 180°.

Based on these structural constraints, the 3D structure of the DNA duplex with T–Hg^II^–T pairs was calculated by simulated annealing, using the program X-PLOR ver 3.851 (40), based on previously reported protocols (41). From the calculations, 17 structures that satisfied the experimental constraints and covalent geometries were obtained out of 100 randomized structures (Supplementary Figure S3). Statistics for the converged structures are shown in Supplementary Table S2. Through the structure calculations, the N–Hg^II^–N linkages of the T–Hg^II^–T pairs were maintained. This is because the pairing partners of each T–Hg^II^–T pair had already been determined in the same DNA sequence from the 2-bond ^15^N–^15^N *J*-coupling across Hg^II^ (^2^*J*_NN_) (23) (Figure 1d).

For further information on the NMR measurements and the structure calculation, see Supplementary Material. The structure is deposited in the Protein Data Bank with ID 2rt8.

### ONIOM QM/QM calculations, structural modelling and geometry optimization

**Figure 3.**
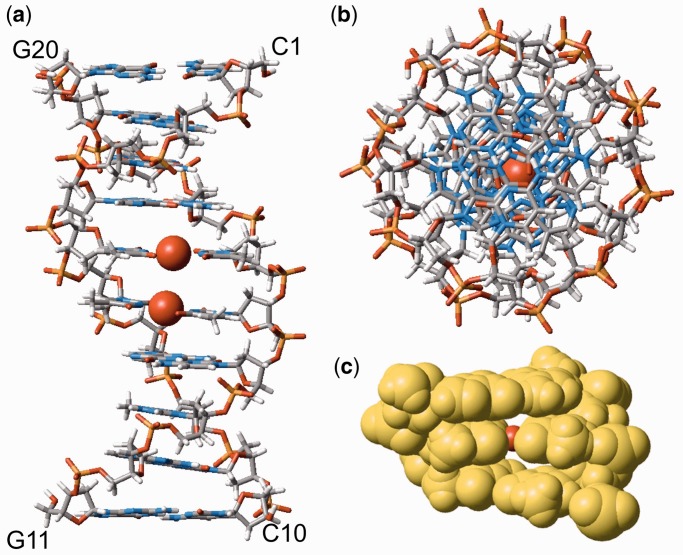
The 3D structure of Hg^II^-bound DNA duplex **1**•**2**. (**a**) The side view perpendicular to helical axis. (**b**) The top view along helical axis. (**c**) The space-filling model of the middle 3-bp DNA segment including G4–C17, T5–Hg^II^–T16 and T6–Hg^II^–T15 pairs sketched out in Figure 1e. The Hg atoms are depicted always as red balls. The Hg–Hg distance derived solely with NOEs was ∼4 Å. When we applied the Hg–Hg distance constraint at 3.3 Å reflecting our X-ray diffraction analysis of a DNA duplex with tandem T–Hg^II^–T base pairs (53) (Supplementary Figure S6 and Supplementary Material), the derived model structure of duplex **1**•**2** (Supplementary Figure S7) was consistent with the NOE constraints.

The structural model employed in the ONIOM QM/QM calculations (42); CAM-B3LYP(6-31G*, Stuttgart ECP for Hg):BP86(LANL2DZ) with GAUSSIAN 09 (43), was derived from the NMR structure of the DNA duplex **1**•**2**, and is schematically depicted in Figure 1e (the G4–C17, T5–Hg^II^–T16 and T6–Hg^II^–T15 base pairs). The implicit water solvent was employed in all calculations. The geometry optimized structures for product and reactant adjusted from Equation (1) in Results and discussion section are depicted in Supplementary Figures S4 and S5. For the derivation of Equation (1), see Supplementary Material. In the reactant, hydrated Hg^II^ bound to the DNA(T–T) while in the DNA(T–Hg^II^–T) product Hg^II^ was completely dehydrated. The overall helical structure of the models was ensured by relevant constraints adopted from the 3D structure of Figure 3; only the middle base pair was geometry optimized (see the legend to Supplementary Figures S4 and S5 for details). The Δ*H*, Δ*S* and Δ*G* were calculated for *T* = 298.15 K and standard pressure within the rigid-rotor harmonic-oscillator approximation, *S* was composed of translation, rotation and vibration contributions. For ONIOM QM/QM calculations, see also Supplementary Material.

### Preliminary X-ray diffraction analysis of the DNA duplex and modelling of the DNA duplex 1•2 with an experimental Hg–Hg distance constraint

To obtain experimental Hg^II^–Hg^II^ distance in a DNA duplex, a preliminary X-ray diffraction analysis was performed. For this purpose, the DNA dodecamer was co-crystallized with Hg(ClO_4_)_2_ by the hanging-drop vapour diffusion method at 4°C. Preliminary X-ray data collections were performed with synchrotron radiation (*λ* = 0.98Å) at BL17A in the Photon Factory (Tsukuba, Japan). Two Hg^II^ atoms were found at coordinates (*x*, *y*, *z*) = (1.1, 0.0, 7.2), (1.6, 0.9, 10.3) (Supplementary Figure S6) using the heavy-atom-search procedure of the program *AutoSol* from the *Phenix* suite (44–46). Then, the Hg^II^–Hg^II^ distance was determined as 3.3Å which is also consistent with its theoretical values, 3.28–3.52Å (30). Based on these facts, the model structure of the DNA duplex with T–Hg^II^–T pairs was also calculated by using rigid body minimizations and following normal energy minimizations under the Hg^II^–Hg^II^ distance constraint (3.3Å). The derived model structure is shown in Supplementary Figure S7. For details on the preliminary X-ray diffraction analysis and the modelling studies, see Supplementary Material.

## RESULTS AND DISCUSSION

### Thermal denaturation experiment

Two non-self-complementary DNA duplexes presented in Figure 1 were chemically synthesized. The thermal stabilities (*T*_m_) of duplex **1**•**2** with T–T mismatches and T–Hg^II^–T base pairs were evaluated and compared with that of reference duplex **1**•**3** containing W–C T–A base pairs (Figure 2).

The *T*_m_ value for duplex **1**•**2** increased from 25°C to 54°C upon adding Hg^II^. Interestingly, the DNA duplex **1**•**2** with T–Hg^II^–T pairs was more stable than its reference duplex **1**•**3** with W–C base pairs. The T–Hg^II^–T base pair is therefore more stable than the W–C base pair in the sequence context of the DNA duplex **1**•**2**. We also studied the effects of T–Hg^II^–T nearest neighbour base pairs on *T*_m_ value (Supplementary Figure S1) and the highest stability was observed for the closely related sequence with the Hg^II^-bound DNA duplex **1**•**2**.

### Structure determination

The DNA duplex **1**•**2** was selected for structure determination because: (i) the chemical structure of T–Hg^II^–T was determined with ^2^*J*_NN_: 2-bond ^15^N–^15^N *J*-coupling across Hg^II^-mediated linkage (Figure 1d) for the same sequence of DNA oligomer (23), and (ii) the closely related sequence was thermally most stable (Supplementary Figure S1). We then recorded NOESY spectra of Hg^II^-bound DNA duplex **1**•**2** (39), and generated distance constraints from NOESY spectra published in the reference (39) (Supplementary Table S2).

In total, 17 structures that satisfied the NOE constraints were obtained (Supplementary Figure S3 and Table S2). All the derived structures were normal B-form duplexes (Supplementary Figure S3). The 3D structure of the duplex with the lowest energy is shown in Figure 3. The T–Hg^II^–T pairs are well stacked without distorting the duplex, which indicates that they are accommodated into the DNA duplex in a similar manner to canonical W–C base pairs.

In addition, due to the local topology of the T–Hg^II^–T pairs, their C1′–C1′ distances are shorter by ∼1Å than those found in W–C base pairs. Nonetheless, the difference was within the structural variation of B-form DNA duplexes. Therefore, the T–Hg^II^–T pairs structurally mimic W–C base pairs without any significant perturbation of the double helix. This fact most likely explains why the DNA polymerase can incorporate thymine (T) against T in the template strand via the formation of T–Hg^II^–T pair (47,48).

Closer look along the helical axis of DNA duplex **1**•**2** revealed perfectly aligned Hg atoms (Figure 3b). The space-filling model of the respective part of DNA duplex **1**•**2** further revealed that Hg atoms are shielded from bulk water (Figure 3c). The well-stacked metallo base pairs with the Hg^II^–Hg^II^ distance at 4.03–4.17Å and narrow O4−O4/O2−O2 spacing exclude any possibility for bulk water to penetrate into proximity of Hg atoms. The relationship between 3D structure and the thermodynamic parameters will be discussed later.

Based on the 3D structure, we confirmed the theoretical prediction by Voityuk (49) and us (29) that overlap of their LUMOs of Hg atoms in the metallo-DNA is possible. This implies that the metallo-DNA duplex could be effective route for (an) excess electron(s). Furthermore, the well-stacked arrangement of T–Hg^II^–T pairs suggests that interaction of Hg atoms is not repulsive, which supports existence of the Hg–Hg metallophilic attraction inside metallo-DNA. Recently, existence of the metallophillic attraction between heavy metals in metallo-DNAs was theoretically proposed for the Ag^I^-mediated imidazole–imidazole base pairs (50) and the T–Hg^II^–T pairs (30,31), and the 3D structure presented here is an additional indicative of such a newly proposed attractive force between heavy metals.

### The relationship between 3D structure and the thermodynamic parameters

Next, we considered relationship between the 3D structure and the thermodynamic parameters for the T–Hg^II^–T formation determined by Torigoe’s group (24,25), which showed positive Δ*S* and negative Δ*H* (Table 1). Based on the 3D structure, the reaction for T–Hg^II^–T base pair formation can be written as follows. See also Supplementary Material for detailed derivation of Equation (1).
(1)
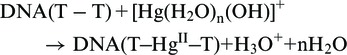

where the DNA(T–T) and DNA(T–Hg^II^–T) stand for DNA duplex with T–T mismatch and that with T–Hg^II^–T pair, respectively (Figure 1e). In the Equation (1), we considered (i) the imino proton (H^+^)–Hg^II^ exchange reaction upon T–Hg^II^–T base pair formation (Figure 1d); (ii) the dehydration of Hg^II^ cation during reaction; and (iii) the p*K*_a_ = 3.4 for Hg^II^–aqua complex (51) that implies existence of hydroxy-ligand of Hg^II^.

**Table 1.** Experimental and theoretical thermodynamic parameters

	Δ*H°*/kcal/mol	Δ*S°*/cal/mol/K	Δ*G°*/kcal/mol	Reference
Experimental (ITC)^a^	−3.85 ± 0.18	13.1 ± 0.65	−7.76 ± 0.19	(24)
	−4.76 ± 0.13	10.6 ± 0.84	−7.91 ± 0.12	(24)
Theoretical^b^	−4.04	14.2	−8.27	This work

^a^In reference (25), thermodynamic parameters possessed much larger standard deviations. Therefore, only the precise data from reference (24) were shown in table. ^b^Calculated values are based on Equation (1) (see Supplementary Figures S4 and S5, and Supplementary Methods). Δ*G°* values are given at 298.15 K.

Upon the Hg^II^-binding to T–T mismatch, number of water molecules initially coordinated to Hg^II^ were released to bulk. Accordingly, the dehydration of Hg^II^ should yield the entropy increase following the thermodynamic assumptions. Such positive Δ*S* is known as dehydration entropy. However, the complete dehydration in this case has been only rarely validated experimentally. In summary, based on the 3D structure, one contributor to the Δ*S* was identified as dehydration entropy owing to the complete dehydration of Hg^II^.

The 3D structure of Hg^II^-bound DNA duplex **1**•**2** enabled calculation of the Δ*S* and Δ*H* with the ONIOM QM/QM method. Using the 3-bp DNA segment (Figures 1e and 3c) derived from the 3D structure of DNA duplex **1**•**2**, the models of product (Supplementary Figure S4) and reactant (Supplementary Figure S5) were constructed following the Equation (1). Based on these structures, the thermodynamic parameters were calculated (Table 1). The calculated Δ*H* (−4.04 kcal/mol) and Δ*S* (14.2 cal/mol/K) agreed with the experiment (Table 1). From the result, not only the absolute values of Δ*H* and Δ*S*, but also the positive sign for Δ*S* was reproduced by theory. In addition, the calculated thermodynamic parameters in this work were consistent with those derived previously with different protocols for the complete reaction pathway describing formation of T–Hg^II^–T base pair (52). The determined 3D structure rationally explained the thermodynamic parameters.

## CONCLUSION

The first 3D structure of metallo-DNA composed exclusively of ‘NATURAL’ bases and containing tandem T–Hg^II^–T base pairs was determined in solution. The positive Δ*S* recorded for T–Hg^II^–T base pair formation was explained as Hg^II^-dehydration entropy on the structural basis. The 3D structure rationally explained the specific Hg^II^ affinity toward T–T mismatch and unveiled the 3D arrangement of the metallo base pairs in the DNA duplex.

## ACCESSION NUMBERS

PDB ID: 2rt8 BioMagResBank accession number: 11528

## SUPPLEMENTARY DATA

Supplementary Data are available at NAR Online.

## FUNDING

Ministry of Education, Culture, Sports, Science and Technology, Japan [grants-in-aid for Scientific Research (B) (24310163 to Y.T.) and (C) (18550146 to Y.T.)]; Human Frontier Science Program Organization, France (Human Frontier Science Program, young investigator grant to Y.T. and V.S.); GAČR (Czech Republic) [P205/10/0228 to V.S.]; Intelligent Cosmos Foundation (to Y.T.); Daiichi-Sankyo Foundation of Life Science and the Invitation Fellowship for Research in Japan (short-term) from the JSPS (to Y.T. and V.S.). Funding for open access charges: Grant-in-aid for Scientific Research (B) [24310163] from the Ministry of Education, Culture, Sports, Science and Technology, Japan.

*Conflict of interest statement*. None declared.

## Supplementary Material

Supplementary Data
