# Supplementary material for: The structure of metallo-DNA with consecutive thymine–HgII–thymine base pairs explains positive entropy for the metallo base pair formation
Source: Nucleic Acids Res. 2013 Dec 26;42(6):4094–9. doi: 10.1093/nar/gkt1344 (PMC3973346; doi:10.1093/nar/gkt1344)
Supplement: Supplementary Data [file supp_42_6_4094__index.html]

The structure of metallo-DNA with consecutive thymine–HgII–thymine base pairs explains positive entropy for the metallo base pair formation — The structure of metallo-DNA with consecutive thymine–HgII–thymine base pairs explains positive entropy for the metallo base pair formation — Supplementary Data 

# The structure of metallo-DNA with consecutive thymine–HgII–thymine base pairs explains positive entropy for the metallo base pair formation

## Supplementary Data

files

**Files in this Data Supplement:**

- Supplementary Data - pdf file
