# Supplementary material for: The structure of metallo-DNA with consecutive thymine–HgII–thymine base pairs explains positive entropy for the metallo base pair formation
Source: Nucleic Acids Res. 2013 Dec 26;42(6):4094–9. doi: 10.1093/nar/gkt1344 (PMC3973346; doi:10.1093/nar/gkt1344)
Supplement: Supplementary Data [file supp_gkt1344_nar-01709-f-2013-File005.pdf]

Supplementary Data

The structure of metallo-DNA with consecutive T-Hg^{II}-T base-pairs explains positive entropy for the metallo-base-pair formation

Hiroshi Yamaguchi[1,‡], Jakub Šebera[2,‡], Jiro Kondo[3,‡], Shuji Oda[1], Tomoyuki Komuro[1], Takuya Kawamura[1], Takenori Dairaku[1], Yoshinori Kondo[1], Itaru Okamoto[4], Akira Ono[4], Jaroslav V. Burda[5], Chojiro Kojima[6], Vladimír Sychrovský[2,*], Yoshiyuki Tanaka[1,*]

[1] Laboratory of Molecular Transformation, Graduate School of Pharmaceutical Sciences, Tohoku University, 6-3 Aza-Aoba, Aramaki, Aoba-ku, Sendai, Miyagi 980-8578, Japan

[2] Institute of Organic Chemistry and Biochemistry, Academy of Sciences, Flemingovo nám. 2, 16610, Praha 6, Czech Republic

[3] Department of Materials and Life Sciences, Faculty of Science and Technology, Sophia University, 7-1 Kioicho, Chiyoda-ku, Tokyo 102-8554, Japan

[4] Department of Material & Life Chemistry, Faculty of Engineering, Kanagawa University, 3-27-1 Rokkakubashi, Kanagawa, Yokohama, Kanagawa-ken 221-8686 Japan

[5] Department of Chemical Physics and Optics, Faculty of Mathematics and Physics, Charles University in Prague, Ke Karlovu 3, 121 16 Prague 2, Czech Republic

[6] Institute for Protein Research, Osaka University, 3-2 Yamadaoka, Suita, Osaka 565-0871, Japan

[‡]These authors are equally contributed to this work.

*Corresponding authors

Contents

Supplementary Methods

Table S1. Assignment table of ^{1}H resonances of Hg^{II}-bound DNA duplex **1•2**

Table S2. Statistics of constraints and calculated structures

Figure S1. The nearest neighbour effect on the tandem T-Hg^{II}-T base-pairs

Figure S2. Concentration dependency of the thermal denaturation profiles of the Hg^{II}-bound DNA duplex **1•2**

Figure S3. Overlay of the 17 converged structures

Figure S4. The DNA molecule employed as the model of the product for obtaining theoretical thermodynamic parameters

Figure S5. The DNA molecule employed as the model of the reactant for obtaining theoretical thermodynamic parameters

Figure S6. Density-modified electron density maps

Figure S7. Model structure of the Hg^{II}-bound DNA duplex **1•2** under the Hg^{II}-Hg^{II} distance constraint of 3.3 Å and NOE constraints

Supplementary Methods

Thermal denaturation experiments. The DNA oligomers shown in Figures 1 (decamer duplexes) and S1 (dodecamer duplexes) were chemically synthesized and purified as described previously (23). All sample solutions for thermal denaturation experiments contained 5.0 μM DNA duplex, 10 mM Na-cacodylate buffer pH 6.0 and 100 mM $NaClO_4$. The sample solution for the Hg^{II}-bound DNA duplexes **1•2** ("**1•2**(T-Hg^{II}-T)" in Figure 2) contained 10.5 μM $HgClO_4$, whereas that for DNA duplexes **1•2** alone ("**1•2**(T-T)" in Figure 2) did not. Since DNA duplex **1•3** ("**1•3**(T-A)" in Figure 2) is a control experiment against Hg^{II}-bound DNA duplex **1•2** ("**1•2**(T-Hg^{II}-T)" in Figure 2), 10.5 μM $HgClO_4$ was added to the sample solution of DNA duplex **1•3** in order to adjust the Hg^{II} concentration against the Hg^{II}-bound DNA duplexes **1•2**. It is also known that Hg^{2+}-addition has a little effect onto thermal denaturation profiles under standard conditions for T_m-measurements (24). UV spectra of these solutions were recorded every 3 °C for "**1•2**(T-Hg^{II}-T)" and "**1•3**(T-A)", and every 2 °C for "**1•2**(T-T)". In the temperature profiles, UV absorbances at 260 nm were plotted against temperature (Figure 2). The T_m values were determined as described in the literature (38).

The T_m values against the nearest neighbour Watson-Crick base-pairs to T-Hg^{II}-T base-pairs were studied by using a dodecamer hetero duplex: d(CCGC**X**TT**V**TCCG) • d(CGGA**W**TT**Y**GCGG), where **X**-**Y** and **V**-**W** are Watson-Crick base-pairs. DNA sequences with all possible 10 combinations of **X**-**Y** and **V**-**W** base-pairs within 4-base-paired unit of 5' XTTV 3' • 5' WTTY 3' were prepared. All the sample solutions for thermal denaturation experiments contained 5.0 μM DNA duplex, 10 mM Na-cacodylate buffer pH 6.0, 100 mM $NaClO_4$ and 10.5 μM $HgClO_4$. UV spectra of these solutions were recorded every 3 °C, and UV absorbances at 260 nm were plotted against temperature in the temperature profiles (Figure S1). The T_m values were determined as described in the literature (38).

The concentration dependent T_m changes are presented in Figure S2. The solutions for this set of experiments contained 10 mM Na-cacodylate pH 6.0, 50 mM $NaClO_4$, and 42 μM (closed circle) and 5.2 μM (open circle) DNA duplex **1•2**. A slight excess amount (1.2 molar equivalents) of Hg^{II} to a T-T mismatch was added to each solution. For high and low concentrations, UV cells with the path-length at 1 mm and 1 cm were used, respectively.

In all the thermal denaturation experiment, we confirmed that temperature profiles for increasing and decreasing temperatures were identical within the experimental error range.

NMR measurements and three-dimensional structure determination. Sample solution for ^1H resonance assignments and the structure determination was the same one as described previously (23). This solution contained 2.0 mM DNA duplex, 1.0 mM Na-cacodylate buffer pH 6.0, 100 mM $NaClO_4$, 1.0 molar equivalents Hg^{II} against the T-T mismatch in 100% D_2O (DQF-COSY) or 95% H_2O/5% D_2O (NOESY). Stoichiometry between Hg^{II} and the T-T mismatch was ensured by chelating excess Hg^{II} as described previously (23).

Two-dimensional ^{1}H-^{1}H DQF-COSY spectrum was recorded with a 8192*1024 complex points against a spectral width of 8013*8013 Hz at 293 K on a Bruker DRX800 spectrometer. Sixteen scans were averaged at each transient. Two-dimensional ^{1}H-^{1}H NOESY spectrum was recorded at 8192*2048 complex points against a spectral width of 17986*18002 Hz at 283 K on a Bruker DRX800 spectrometer. Sixteen scans were averaged at each transient.

Using the NMR spectra, we were able to assign all the non-exchangeable protons (39) and most of exchangeable protons. The complete assignments are reported in Table S1, and deposited in the Biological Magnetic Resonance Data Bank with accession number 11528.

Volumes of the NOE cross peaks were quantitated by Sparky (53). Peak volumes were converted into distance constraints (Table S2) by using the program Mardigras (54,55). The derived distance constraints were subjected to the 3-dimensional (3D) structural calculation of the DNA duplex in complex with Hg^{II}. For other constraints, torsion angle constraints for a C2'-*endo* sugar puckering (Table S2) were applied except for the T15 residue. This puckering selection was based on results in a DQF-COSY spectrum where canonical patterns of H1'-H2'/H2" cross peaks for the C2'-*endo* sugar puckering were observed except for the T15 residue (39). Therefore, no constraints for sugar puckering were applied to the T15 residue. For Watson-Crick base-pairs and T-Hg^{II}-T base-pairs, planarity constraints for the base-pairs were applied (Table S2). The structural constraints for T-Hg^{II}-T base-pairs were generated based on the crystal structure of the 1-methylthymine-Hg^{II} (2:1) complex (37), i.e., N3-Hg^{II} bond length: 2.04 Å and N3-Hg^{II}-N3 bond angle: 180°.

Based on these structural constraints, the 3D structure of the DNA duplex with T-Hg^{II}-T base-pairs was calculated by simulated annealing, using the program X-PLOR ver 3.851 (40). Initially, 100 independent randomized structures were generated by molecular dynamics at 350 K, as reported previously (41). In the simulated annealing, the high temperature was set at 3000 K with all atomic mass units of 100 amu (default), and the temperature was decreased to 300 K by 50 K steps. After the energy minimization steps, 17 structures that satisfied the experimental constraints and covalent geometries were obtained (Figure S3). Statistics for the converged structures are also shown in Table S2. Through the structure calculations, the N-Hg^{II}-N linkages of the T-Hg^{II}-T pairs were maintained. This is because the pairing partners of each T-Hg^{II}-T base-pair had already been determined in the same DNA sequence from the 2-bond ^{15}N-^{15}N *J*-coupling across Hg^{II} ($^{2}J_{NN}$) (23) (Figure 1d). The structure is deposited in the Protein Data Bank with ID 2rt8.

The Hg^{II}-Hg^{II} distance ~4 Å within the determined 3D structures (Figures 3 and S3) was longer than the normal distance between stacked base-pairs in B-form DNA (3.4 Å). However, this may be due to sparse NOE distance constraints around the T-Hg^{II}-T pairs, and modelling of the Hg^{II}-bound DNA duplex **1•2** with an additional Hg^{II}-Hg^{II} distance constraints at 3.3 Å was performed (see Figure S7 and the next section). The Hg^{II}-Hg^{II} distance at 3.3 Å was suggested from a preliminary X-ray diffraction analysis of the DNA

duplex with tandem T-Hg^{II}-T base-pairs (see Figure S4 and the next section) and the recent theoretical study of T-Hg^{II}-T pairs that focused on metallophilic attraction (30).

Preliminary X-ray diffraction analysis of the DNA duplex and modelling of the DNA duplex 1•2 with an experimental Hg-Hg distance constraint. To obtain experimental Hg^{II}-Hg^{II} distance in a DNA duplex, a preliminary X-ray diffraction analysis was performed. A DNA dodecamer with the sequence d(CGCGATTTCGCG) was chemically synthesized by FASMAC (Japan), purified by 20% polyacrylamide gel electrophoresis under denaturing conditions and desalted by ultrafiltration. The DNA dodecamer was co-crystallized with $Hg(ClO_4)_2$ by the hanging-drop vapour diffusion method at 4 °C. Single crystals were obtained at several conditions containing 50 mM sodium cacodylate (pH 7.0), 5−100 mM KCl, 1−10 mM spermine tetrahydrochloride and 1−10% 2-methyl-2,4-pentanediol. Preliminary X-ray data collections were performed with synchrotron radiation (λ = 0.98 Å) at BL17A in the Photon Factory (Tsukuba, Japan). One of these crystals diffracted to 2.6 Å. Two Hg^{2+} ions were found at coordinates (x, y, z) = (1.1, 0.0, 7.2), (1.6, 0.9, 10.3) (Figure S4) using the heavy-atom search procedure of the program *AutoSol* from the *Phenix* suite (44-46), which was confirmed by a determined crystal structure at the later stage (J.K. personal communication). The distance between the two Hg^{II} atoms is 3.3 Å, which is about the same distance observed between two base-pairs in a DNA helix. The derived experimental distance (3.3 Å) is consistent with the recent theoretical values (3.28−3.52 Å), which focused on the metallophilic attraction (30).

Based on these facts, the model structure of the DNA duplex with T-Hg^{II}-T pairs was also calculated by using rigid body minimizations and following normal energy minimizations under the Hg^{II}-Hg^{II} distance constraint (3.3 Å). The structural modelling of the DNA duplex with T-Hg^{II}-T base-pairs under the Hg^{II}-Hg^{II} distance constraint (3.3 Å) was performed using the program X-PLOR ver 3.851 (40). In the initial stage, we performed rigid body minimizations using the Hg^{II}-Hg^{II} distance and the NOE distance constraints as target functions by dividing the DNA duplex into left and right segments, as shown in Figure 1b. To eliminate the distortion of the covalent geometry after rigid body minimization, normal energy minimizations were applied only to the backbone of the T-Hg^{II}-T base-pairs with the other parts of the duplex fixed.

The calculated model structure of the Hg^{II}-bound DNA duplex 1•2 with the additional distance constraint was derived without any deviation from the experimental constraints (Figure S7). It is consistent with an observation that the sequential NOE walks were traceable, even around the tandem T-Hg^{II}-T pairs (39), which implies that these base-pairs stack in a similar manner to W-C base-pairs. The overlap of LUMO orbitals for neighbouring T-Hg^{II}-T pairs may have to be considered based on normal base-pair distances in B-form DNA.

ONIOM QM/QM calculations, structural modelling and geometry optimization. The structural model employed in the ONIOM QM/QM calculations (42); CAM-B3LYP(6-31G*, Stuttgart ECP for Hg):BP86(LANL2DZ) with GAUSSIAN 09 (43), was derived from the NMR structure of the DNA duplex **1•2**, and is schematically depicted in Figure 1e (the G4-C17, T5-Hg^{II}-T16 and T6-Hg^{II}-T15 base-pairs). The implicit water solvent was employed in all calculations. The geometry optimized structures for reactant and product adjusted from eq. 1 are depicted in Figures S5 and S6 (see the next section for a derivation of eq. 1). In the reactant, hydrated Hg^{II} bound to the DNA(T-T) while in the DNA(T-Hg^{II}-T) product Hg^{II} was completely dehydrated. The overall helical structure of the models was ensured by relevant constraints adopted from the 3D structure of Figure 3; only the middle base-pair was geometry optimized (see also the legend to Figures S5 and S6). The ΔH, ΔS and ΔG were calculated for T = 298.15 K and standard pressure within the rigid-rotor harmonic-oscillator approximation, *S* was composed of translation, rotation and vibration contributions.

Based on the reactant (Figures S6) and product (Figures S5) adjusted from eq. 1, the thermodynamic parameters in Table 1 were calculated. The calculated ΔH (–4.04 kcal mol^{-1}) and ΔS (14.2 cal mol^{-1} K^{-1}) agreed with the experimental data (24) (Table 1) with respect to their absolute values. More importantly, the positive sign for the reaction entropy was reproduced by theory. We can therefore conclude that the positive ΔS results both from mercury dehydration (validated by the 3D structure in this work) and changes in the chemical bonding (theoretical calculation of the complete reaction pathway in reference-52).

Equilibrium system for the T-Hg^{II}-T base-pair formation The chemical reaction describing the formation of the T-Hg^{II}-T base-pair can be written as an imino proton (H^+)-Hg^{II} exchange reaction (Figure 1d). The simplest expression of such a reaction is as follows.

$$\text{DNA(T-T)} + Hg^{2+} + 2H_2O \rightarrow \text{DNA(T-}Hg^{II}\text{-T)} + 2H_3O^+ \quad \text{(eq. S1)}$$

where DNA(T-T) and DNA(T-Hg^{II}-T) represent a DNA duplex with a T-T mismatch and a T-Hg^{II}-T base-pair, respectively (Figure 1e). Here, the Hg^{2+} ions must be considered with caution as they are hydrated and form aqua-complexes $[Hg(H_2O)_m]^{2+}$ in solution. In addition, the formation of the T-Hg^{II}-T base pair proceeds together with complete dehydration of Hg^{2+}, as clearly observed in the 3D structure (Figure 3c). Considering the hydration/dehydration of Hg^{2+} during the reaction, the scheme should be modified as follows.

$$\text{DNA(T-T)} + [Hg(H_2O)_m]^{2+} \rightarrow \text{DNA(T-}Hg^{II}\text{-T)} + 2H_3O^+ + (m-2)H_2O \qquad \text{(eq. S2)}$$

If we further consider the pK_a of the aqua-complex of Hg^{2+} (pK_a 3.4 (51)), eq. S2 should be rewritten as follows. (Under neutral pH, the $[Hg(H_2O)_n(OH)]^+$ would be the dominant form of the aqueous complex of Hg^{2+}.)

$$\text{DNA(T-T)} + [Hg(H_2O)_n(OH)]^+ \rightarrow \text{DNA(T-}Hg^{II}\text{-T)} + H_3O^+ + nH_2O \qquad \text{(eq. 1)}$$

Therefore, the equilibrium system presented in eq. 1 was used to estimate the thermodynamic parameters in the text.

53. Goddard, T. D. and Kneller, D. G. SPARKY 3, University of California, San Francisco.

54. Borgias, B. A. and James, T. L. Two-dimensional nuclear Overhauser effect - complete relaxation matrix analysis. Methods Enzymol., 176, 169-183 (1989).

55. Borgias, B. A. and James, T. L. MARDIGRAS - A procedure for matrix analysis of relaxation for discerning geometry of an aqueous structure. J. Magn. Reson., 87, 475-487 (1990).

Table S1 Assignment table of ^{1}H resonances of Hg^{II}-bound DNA duplex 1•2[a].

	H6/H8	H2/H5/Me	H1'	H2'	H2"	H3'	H4'	H5'	H5"	amino protons		imino proton
C1	7.61	5.89	5.74	1.94	2.38	4.69	4.05	3.70	3.70	7.72	6.56	n.a
G2	7.96	n.a	5.86	2.66	2.69	4.96	4.33	3.96	4.07	8.47	6.99	13.11
C3	7.34	5.44	5.72	2.01	2.43	4.85	4.18	4.13	4.18	8.36	6.56	n.a
G4	7.98	n.a	6.03	2.58	2.82	4.95	4.39	4.09	4.12	8.33	7.22	13.61
T5	7.06	1.56	5.82	1.89	2.51	4.75	4.12	4.09	4.27	n.a	n.a	n.a
T6	7.38	1.80	5.76	2.22	2.27	4.82	3.99	4.08	4.10	n.a	n.a	n.a
G7	7.87	n.a	6.02	2.64	2.81	4.92	4.35	3.97	4.08	–	6.80	12.64
T8	7.28	1.26	6.14	2.20	2.54	4.90	4.23	4.15	4.17	n.a	n.a	13.63
C9	7.58	5.71	6.09	2.21	2.48	4.82	4.16	4.05	4.11	8.49	7.00	n.a
C10	7.67	5.80	6.24	2.27	2.27	4.56	4.04	3.93	4.15	8.31	7.19	n.a
G11	7.81	n.a	5.62	2.45	2.62	4.79	4.13	3.62	3.62	8.38	6.51	12.89
G12	7.82	n.a	5.52	2.67	2.73	4.99	4.34	4.02	4.10	8.07	6.84	12.93
A13	8.17	8.01	6.20	2.66	2.84	5.03	4.46	4.16	4.21	8.21	6.59	n.a
C14	7.38	5.24	5.86	2.29	2.50	4.67	4.28	4.07	4.23	8.32	6.86	n.a
T15	7.20	1.72	5.78	1.72	2.27	4.62	3.92	4.04	4.23	n.a	n.a	n.a
T16	7.56	1.64	6.03	2.25	2.50	4.84	4.16	3.91	3.98	n.a	n.a	n.a
C17	7.50	5.66	5.70	2.11	2.38	4.82	4.08	4.10	4.21	7.75	7.06	n.a
G18	7.90	n.a	5.90	2.61	2.71	4.97	4.34	4.00	4.10	8.34	6.91	12.87
C19	7.32	5.42	5.76	1.90	2.32	4.81	4.15	4.07	4.09	8.51	6.66	n.a
G20	7.94	n.a	6.15	2.36	2.61	4.67	4.17	4.05	4.04	7.25	6.95	12.87

[a] Chemical shifts are reported in ppm. n.a.: Not Applicable (Assignments are not applicable due to the absence of the corresponding protons.). –: The resonance of the corresponding proton was not observed, probably due to the overlap of the signals.

Table S2 Statistics of constraints and calculated structures.

distance constraints based on NOE peak volumes	
total	628
NOEs/residue	31.4
dihedral angle constraints	
C2'-endo sugar puckering	57 (19 residues except for T15)
amino proton orientation	30 (all A, G, C residues)
empirical restraints for the W-C base-pair	
distance restraints	55
planarity	all base-pairs
violations	
NOE constraints > 0.4 Å	none
dihedral angle constraints > 5.0°	none
deviations from standard values (all atoms)[a]	
bond length	0.0094 Å (0.000047)[b]
bond angle	1.9° (0.0044)[b]
improper angle	1.22° (0.0064)[b]
RMSD from mean structure	
all atom	0.84 Å
all heavy atom	0.83 Å

[a] Deviations from standard values for bond lengths, bond angles and improper angles were calculated for converged structures, and the derived values were averaged over those from converged structures. [b] Values in parentheses are standard deviations from the average values.

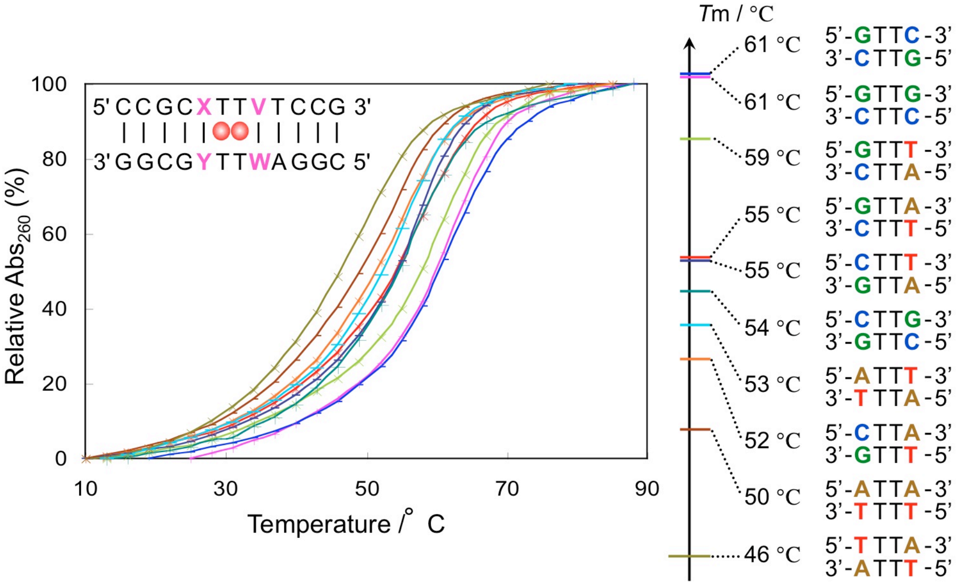

Figure S1 The nearest neighbour effect on the tandem T-Hg^{II}-T base-pairs. Temperature profiles of the UV absorbance for each of the Hg^{II}-bound DNA duplex was examined. The central four base-pair sequences are indicated on the right side of the figure with their T_m values of the corresponding Hg^{II}-bound DNA duplexes. A caution must be paid to interpret the nearest neighbor effect from T_m values, since T_m values include the stability from stacking of the X-Y/V-W and the Watson-Crick base-pairs at outer sides of the four-base-paired unit (5' XTTV 3' • 5' WTTY 3').

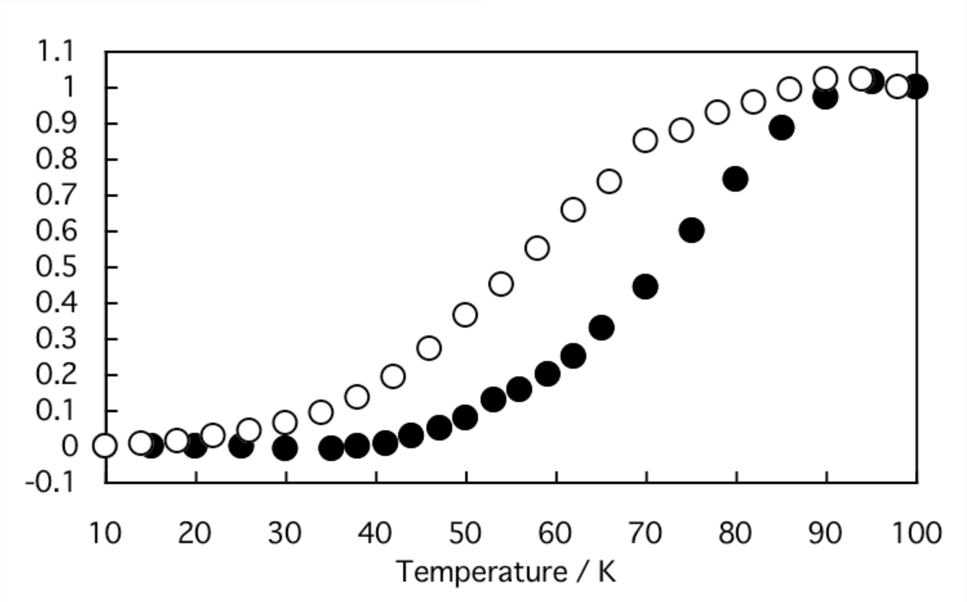

Figure S2 Concentration dependency of the thermal denaturation profiles of the Hg^{II}-bound DNA duplex 1•2. The solution contained 10 mM Na-cacodylate pH 6.0, 50 mM $NaClO_4$, and 42 μM (closed circle) or 5.2 μM (open circle) DNA duplex **1•2**. For each solution, 1.2 molar equivalents Hg^{II} to the T-T mismatch were added. For high and low concentrations, UV cells with a path-length of 1 mm and 1 cm were used, respectively. Note, the T_m value of the duplex **1•2** was dependent on the concentration of the Hg^{II}- bound DNA duplex **1•2**. The formation of T-Hg^{II}-T base-pairs was therefore reversible, in accord with our previous report (29).

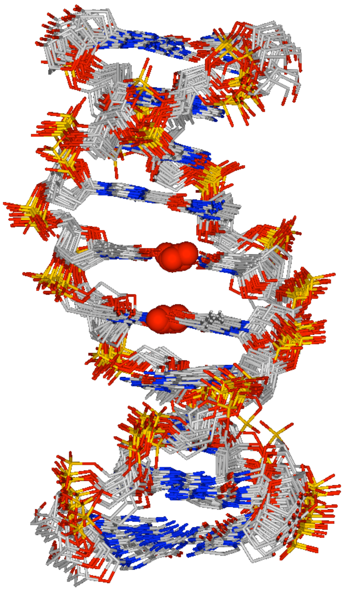

Figure S3 Overlay of the 17 converged structures.

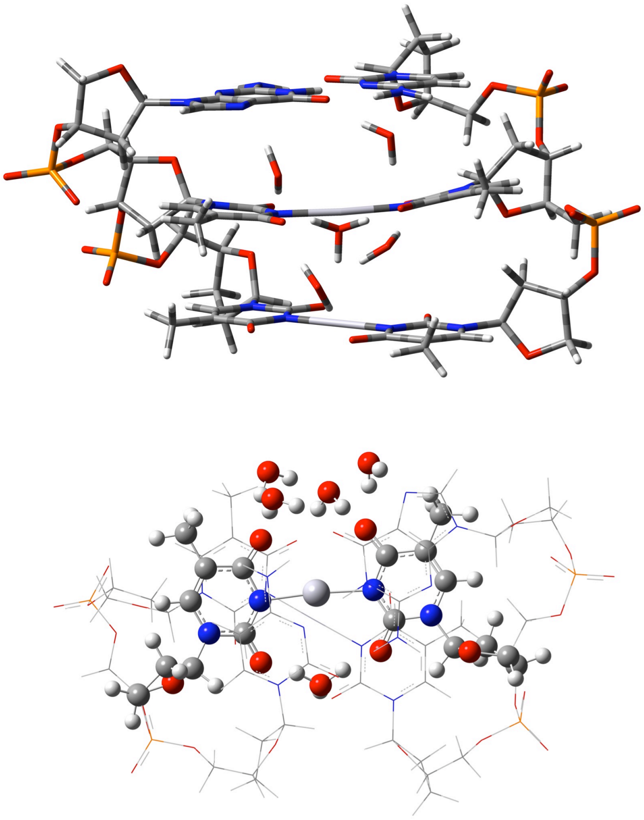

Figure S4 The DNA molecule employed as the model of the product for obtaining theoretical thermodynamic parameters. The side view (top) and the view along the helical axis (bottom). The model duplex contained G4-C17, T5-Hg^{II}-T16 and T6-Hg^{II}-T15 base-pairs of the original DNA duplex **1•2**. The initial geometry employed for geometry optimization with the ONIOM CAM-B3LYP(6-31G*, Stuttgart ECP for Hg):BP86(LANL2DZ) method was derived from the NMR structure solved in this work. The geometry optimization involved only the middle T-Hg^{II}-T base-pair and $[(H_2O)_4(H_3O)]^+$ molecules of solvent depicted in the bottom picture, which defined the small model within the ONIOM method. The rest of the DNA molecule, including the backbone and terminal base-pairs, was fixed during geometry optimization.

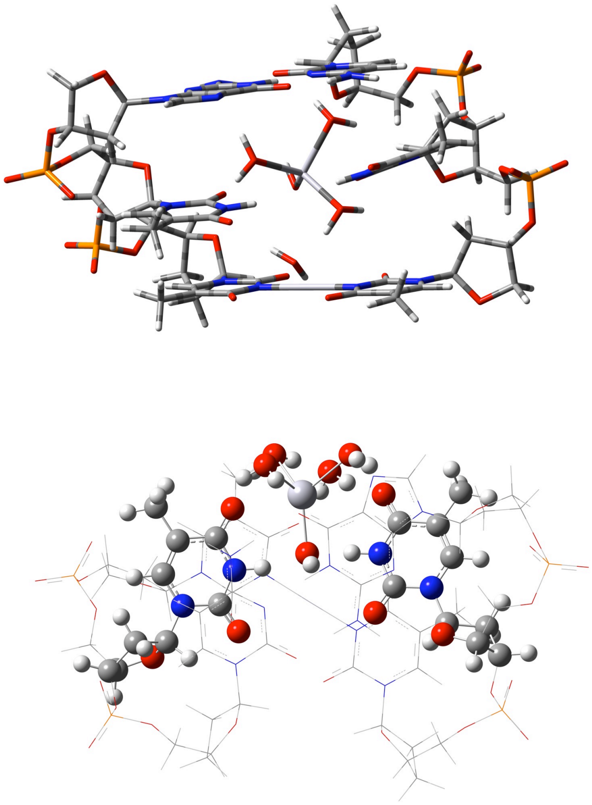

Figure S5 The DNA molecule employed as the model of the reactant for obtaining theoretical thermodynamic parameters. The side view (top) and the view along the helical axis (bottom). The model duplex contained G4-C17, T5-Hg^{II}-T16 and T6-Hg^{II}-T15 base-pairs of the original DNA duplex <u>**1**</u>•<u>**2**</u>. The initial geometry employed for geometry optimization with the ONIOM CAM-B3LYP(6-31G*, Stuttgart ECP for Hg):BP86(LANL2DZ) method was derived from the NMR structure solved in this work as follows. The original T5-Hg^{II}-T16 base-pair was replaced by the T5-T16 mismatch and the $[Hg^{II}(H_2O)_4(HO)]^+$ was coordinated to DNA within its major groove. The hydroxy-ligand of mercury was hydrogen-bonded with the imino protons in the T-T mismatch. The geometry optimization involved only the middle T-T mismatch and the $[Hg^{II}(H_2O)_4(HO)]^+$ cluster depicted in the bottom picture, which defined the small model within the ONIOM method. The rest of the DNA molecule, including the backbone and terminal base-pairs, was fixed during geometry optimization.

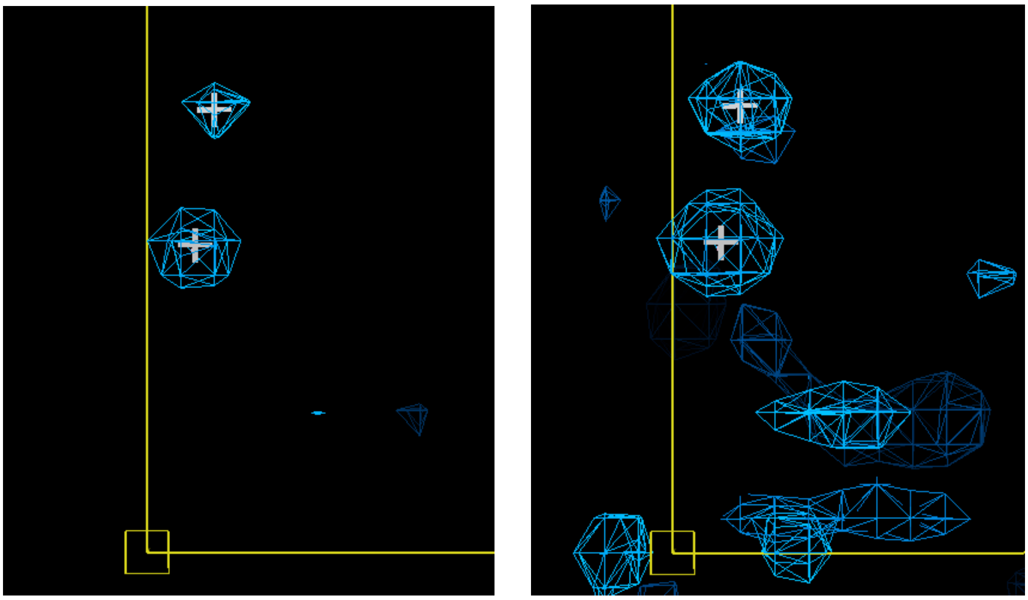

Figure S6 Density-modified electron density maps. They were calculated using the heavy-atom search procedure of the program *AutoSol* from the *phenix* suite contoured at 5σ (left) and 3σ (right). Two Hg^{2+} ions were found at coordinates (*x*, *y*, *z*) = (1.1, 0.0, 7.2) and (1.6, 0.9, 10.3).

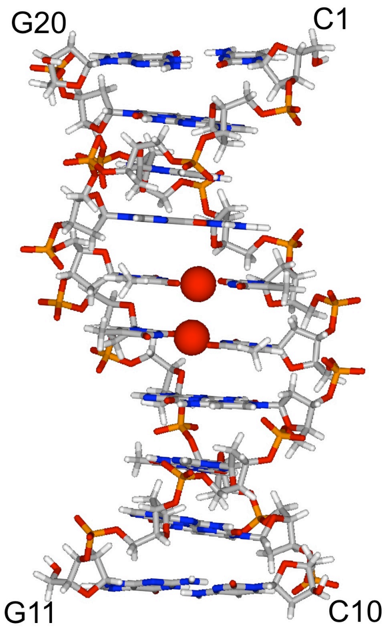

Figure S7 Model structure of the Hg^{II}-bound DNA duplex <u>1</u>•<u>2</u> under the Hg^{II}-Hg^{II} distance constraint of 3.3 Å and NOE constraints.
